# Development and internal validation of machine learning–based models for predicting admission hypothermia in preterm infants: a retrospective cohort study

**DOI:** 10.3389/fmed.2026.1811064

**Published:** 2026-03-25

**Authors:** Xiaokuan Cao, Zuqin Peng, Shihan Yao, Zhengxi Liu, Yanbin Chang

**Affiliations:** Department of Neonatology, Children’s Diagnosis and Treatment Center, The Affiliated Traditional Chinese Medicine Hospital, Southwest Medical University, Luzhou, Sichuan, China

**Keywords:** admission hypothermia, machine learning, neonatal care, preterm infants, risk prediction

## Abstract

**Background:**

Admission hypothermia remains a frequent and preventable complication in preterm infants and is associated with increased morbidity and mortality. Early risk stratification may enable timely thermal management and targeted preventive strategies. This study aimed to develop and internally validate multivariable machine learning–based models for predicting admission hypothermia in preterm infants.

**Methods:**

We conducted a retrospective cohort study including consecutively admitted preterm infants (<37 weeks’ gestation) at a tertiary neonatal referral center in Southwest China (January 2017–January 2025). Admission hypothermia was defined as an axillary temperature <36.5 °C at NICU admission. The dataset was randomly divided into a training cohort (70%) and a validation cohort (30%). Candidate predictors were selected using least absolute shrinkage and selection operator (LASSO) regression. Six models—logistic regression, decision tree, random forest, support vector machine, artificial neural network, and naïve Bayes—were developed. Model performance was evaluated using discrimination (AUC), calibration, Brier score, and classification metrics. Shapley Additive Explanations (SHAP) were applied to enhance interpretability.

**Results:**

Among 346 preterm infants, 154 (44.5%) experienced admission hypothermia. LASSO identified 11 predictors, including gestational age, birth weight, ambient temperature, transport time, inborn status, and preheated incubator use. In the validation cohort, AUCs ranged from 0.78 to 0.86, with logistic regression and artificial neural network demonstrating the highest discrimination (AUC = 0.86). Logistic regression showed favorable calibration and interpretability. SHAP analysis identified lower gestational age, lower birth weight, lower ambient temperature, and longer transport time as the strongest contributors to risk.

**Conclusion:**

Machine learning–based models using routinely available perinatal and environmental variables can effectively predict admission hypothermia in preterm infants. Logistic regression provided robust performance with strong interpretability, supporting its potential integration into early neonatal risk stratification and targeted thermal management strategies.

## Introduction

Preterm birth remains a major global public health challenge and is a leading cause of neonatal morbidity and mortality worldwide (1). Owing to immature thermoregulatory mechanisms, limited subcutaneous fat, a high surface area–to–body mass ratio, and reduced capacity for heat production, preterm infants are particularly vulnerable to hypothermia during the immediate postnatal period (2, 3). Admission hypothermia, commonly defined as a body temperature below 36.5 °C upon arrival at the neonatal unit, has been consistently reported across diverse healthcare settings, even in facilities with established thermal care protocols (4, 5).

A growing body of evidence has demonstrated that admission hypothermia in preterm infants is associated with a wide range of adverse outcomes, including respiratory distress, metabolic acidosis, hypoglycemia, intraventricular hemorrhage, late-onset sepsis, and increased mortality (6–8). Large multicenter cohort studies and international neonatal networks have shown a dose–response relationship between decreasing admission temperature and escalating risks of morbidity and death, particularly among very preterm and very low birth weight infants (9, 10). Consequently, the prevention of admission hypothermia has been emphasized as a core component of neonatal resuscitation and early postnatal care in international guidelines issued by the World Health Organization and professional neonatal societies (11, 12).

Despite these recommendations, admission hypothermia remains prevalent, suggesting that guideline adherence alone may be insufficient to fully mitigate risk. Previous studies have identified multiple factors associated with admission hypothermia, including lower gestational age, lower birth weight, outborn status, prolonged transport time, low ambient temperature, and insufficient use of thermal protection measures (13, 14). However, most existing studies have focused on isolated risk factors or traditional regression-based analyses, which may not adequately capture complex, nonlinear relationships or interactions among clinical, environmental, and transport-related variables. Moreover, few studies have attempted to integrate these routinely available factors into individualized risk prediction tools that could support early identification and targeted intervention in clinical practice.

In recent years, machine learning approaches have gained increasing attention in neonatal research due to their ability to model high-dimensional data and complex relationships without prespecified assumptions (15). Compared with conventional statistical methods, machine learning–based models may offer improved predictive performance and flexibility, particularly in multifactorial conditions such as admission hypothermia. However, evidence regarding the application, validation, and interpretability of machine learning models for predicting admission hypothermia in preterm infants remains limited, and comparative evaluations across different modeling approaches are scarce.

Therefore, the present study aimed to develop and internally validate multivariable machine learning–based prediction models for admission hypothermia in preterm infants using routinely collected clinical, environmental, and transport-related data from a tertiary neonatal referral center. In addition, we sought to compare the performance of multiple machine learning algorithms, identify the most suitable model for clinical application, and enhance interpretability through Shapley Additive Explanations (SHAP) analysis. By providing an interpretable and robust prediction framework, this study seeks to facilitate early risk stratification and support targeted thermal management strategies for preterm infants at the time of hospital admission.

## Materials and methods

### Study design and setting

This retrospective observational cohort study was conducted at a tertiary neonatal referral center in Southwest China between January 2017 and January 2025, with the aim of developing and validating a multivariable prediction model for admission hypothermia in preterm infants. A total of 346 consecutively admitted preterm infants who met the eligibility criteria were included in the analysis. To enable model development and internal validation, the study population was randomly divided into a training cohort and a validation cohort at a ratio of 7:3. All eligible infants admitted to the neonatal unit during the study period were screened, and routine clinical care followed standardized neonatal resuscitation and thermal management protocols in accordance with national and international guidelines. This study is reported in accordance with the TRIPOD + AI statement for prediction model studies using regression or machine learning methods (16, 17).

### Participants

All consecutively admitted preterm infants with a gestational age of less than 37 completed weeks were considered eligible for inclusion if they were admitted to the neonatal unit shortly after birth and had a documented axillary temperature measurement recorded at admission. Infants were excluded if admission axillary temperature (the primary outcome) was not documented in the electronic medical record, if clinical records were implausible or clearly erroneous, or if essential predictor variables required for model development were incomplete. In our center, admission temperature is a mandatory component of standardized NICU admission documentation, and missingness was uncommon during the study period. No additional exclusion criteria related to disease severity, clinical condition, or treatment decisions were applied in order to minimize selection bias and to ensure that the study population reflected routine clinical practice.

### Variables

The primary outcome was admission hypothermia, defined as an axillary temperature <36.5 °C measured at hospital admission, in accordance with World Health Organization recommendations. Candidate predictors were selected *a priori* based on clinical relevance, previous literature, and data availability, and included neonatal characteristics (e.g., gestational age, birth weight, sex), maternal factors (e.g., maternal age), perinatal conditions (e.g., multiple birth, inborn status), environmental exposure [e.g., outdoor ambient temperature at the receiving hospital at admission (°C)], transport-related factors [e.g., transport time (minutes; from departure from the birth/referring location to NICU admission)], and early clinical interventions (e.g., resuscitation, surfactant therapy, endotracheal intubation, use of thermal care measures).

### Data sources and measurement

All data were extracted from the hospital’s electronic medical record system by trained investigators using a standardized data collection form. Gestational age was determined based on obstetric assessment. Birth weight and temperature measurements were obtained using calibrated clinical equipment according to routine practice. Environmental and transport-related variables were recorded at the time of admission. Transport time was defined as the elapsed time (minutes) from departure from the birth/referring location to arrival at the receiving NICU (admission timestamp) and was extracted from the transport/admission record. For prospective implementation, this variable can be entered as the expected/estimated transport duration available before departure based on the referring location and standard transfer workflow (e.g., based on historical median transfer times for each referring site or ambulance dispatch estimates). Categorical variables reflected documented clinical decisions or interventions. Specifically, outdoor ambient temperature at admission was defined as the outdoor temperature (°C) displayed/recorded by the hospital’s weather monitoring system and time-matched to each infant’s NICU admission timestamp. The temperature values were extracted from the electronic record and entered into the analytic dataset.

### Bias

To reduce selection bias, all eligible infants during the study period were consecutively included. Information bias was minimized through standardized data extraction procedures and the use of routinely collected clinical variables. Predictor selection was performed using penalized regression within the training cohort only to limit overfitting, and the validation cohort was kept fully independent to reduce optimism and prevent information leakage. Model performance in the training cohort was assessed using internal cross-validation, and generalizability was evaluated in an independent validation cohort.

### Study size

The final study cohort comprised 346 preterm infants, of whom 154 (44.5%) experienced admission hypothermia (axillary temperature <36.5 °C) at admission. To justify the adequacy of the sample size for multivariable prediction modeling, we calculated the events-per-variable (EPV) for the final parsimonious model using the number of outcome events divided by the number of predictors retained for model development. LASSO feature selection identified 11 candidate predictors that were subsequently used to develop the models; therefore, the overall EPV was 154/11 = 14.0, which meets commonly recommended EPV considerations for minimizing model overfitting in logistic regression–based prediction modeling. In addition, the risk of optimism was further reduced by penalized feature selection (LASSO with cross-validation) and internal validation using both 10-fold cross-validated out-of-fold predictions in the training cohort and performance assessment in an independent validation cohort. Because we also evaluated more flexible algorithms (e.g., ANN/RF/SVM), we additionally relied on cross-validated out-of-fold predictions and a held-out validation cohort to reduce optimism; nevertheless, we acknowledge that internal validation in a single-center split dataset cannot fully exclude residual overfitting, and external validation is therefore essential.

### Quantitative variables

Continuous variables were retained on their original scales whenever possible to preserve information. Variables with skewed distributions were summarized using medians and interquartile ranges. No arbitrary categorization of continuous predictors was performed during model development unless clinically justified.

### Statistical methods

Baseline characteristics were summarized using mean ± standard deviation, median (interquartile range), or number (percentage), as appropriate. Group comparisons were performed using the Student’s t test or Mann–Whitney *U* test for continuous variables and the chi-square test or Fisher’s exact test for categorical variables. The dataset was randomly split prior to any feature selection or model development into a training cohort (70%) and an independent validation cohort (30%). All feature selection, model training, and hyperparameter tuning procedures were conducted exclusively in the training cohort to prevent information leakage. The adequacy of the sample size for prediction modeling was evaluated using events-per-variable (EPV), calculated as the number of admission hypothermia events divided by the number of predictors retained for model development. In the training cohort, least absolute shrinkage and selection operator (LASSO) regression with 10-fold cross-validation was applied to select candidate predictors based on the minimum cross-validated deviance (λ_min). The predictors retained by LASSO were then fixed and carried forward unchanged for model development, and final model performance was evaluated in the independent validation cohort. Selected variables were subsequently used to construct multiple prediction models, including logistic regression, decision tree, random forest, support vector machine, artificial neural network, and naïve Bayes models. Model discrimination was evaluated using the area under the receiver operating characteristic curve (AUC). Calibration was assessed using calibration plots and the Brier score. Accuracy, sensitivity, specificity, and F1 score were calculated using the optimal cutoff determined in the training cohort. Internal performance estimates in the training cohort were derived from out-of-fold predictions generated during cross-validation, while final model performance was assessed in the independent validation cohort. All statistical analyses were performed using R software (version 4.3.2; R Foundation for Statistical Computing, Vienna, Austria), with relevant packages for regression modeling, machine learning, and model evaluation. Reporting items were checked against the TRIPOD + AI checklist (16, 17).

## Results

### Baseline characteristics of preterm infants with and without admission hypothermia

A total of 346 preterm infants were included in the analysis, of whom 192 (55.5%) had no admission hypothermia and 154 (44.5%) experienced admission hypothermia (Table 1). Infants in the hypothermia group had a significantly lower gestational age than those without hypothermia (30.47 ± 2.41 vs. 33.08 ± 1.91 weeks, *p* < 0.001) and a markedly lower birth weight (median 1620.5 g [IQR, 1198.5–1879.8] vs. 2038.5 g [IQR, 1769.8–2377.3], *p* < 0.001) (Table 1). Apgar scores at both 1 min and 5 min were significantly lower among infants with admission hypothermia (both *p* < 0.05). The hypothermia group also had a longer transport time to admission (median 26.0 vs. 22.0 min, *p* = 0.009) and was exposed to a lower ambient temperature at admission (16.20 ± 6.59 vs. 19.43 ± 5.90 °C, *p* < 0.001). Inborn status was less common in the hypothermia group (71.4% vs. 82.3%, *p* = 0.023), whereas resuscitation at birth and surfactant therapy were more frequently required (*p* = 0.047 and *p* = 0.016, respectively). No significant differences were observed between groups with respect to maternal age, sex, multiple birth, mode of delivery, antenatal steroid exposure, maternal comorbidities, or most thermal care practices. Seasonal distribution differed significantly between groups (*p* = 0.001), with a higher proportion of hypothermia cases occurring in winter (Table 1).

**Table 1:** Baseline characteristics of preterm infants with and without admission hypothermia.

Item	Overall (*n* = 346)	No hypothermia (*n* = 192)	Hypothermia (*n* = 154)	*p-*value
Gestational age, weeks^#^	31.92 ± 2.51	33.08 ± 1.91	30.47 ± 2.41	<0.001
Birth weight, g^#^	1863.50 (1488.25–2209.00)	2038.50 (1769.75–2377.25)	1620.50 (1198.50–1879.75)	<0.001
Maternal age, years^#^	29.66 ± 4.72	29.39 ± 4.91	30.01 ± 4.47	0.224
Apgar score at 1 min	6.73 ± 1.67	6.97 ± 1.58	6.42 ± 1.73	0.002
Apgar score at 5 min	8.38 ± 1.61	8.56 ± 1.47	8.16 ± 1.75	0.022
Transport time, min^#^	24.00 (15.00–35.00)	22.00 (14.00–32.00)	26.00 (17.00–39.00)	0.009
Ambient temperature, °C^#^	17.99 ± 6.41	19.43 ± 5.90	16.20 ± 6.59	<0.001
Time to first temperature measurement, min^#^	15.00 (11.00–19.75)	15.00 (11.00–19.00)	16.00 (10.25–20.00)	0.484
Male sex	179 (51.7)	101 (52.6)	78 (50.6)	0.800
Multiple birth^#^	82 (23.7)	42 (21.9)	40 (26.0)	0.445
Cesarean delivery	167 (48.3)	92 (47.9)	75 (48.7)	0.971
Inborn^#^	268 (77.5)	158 (82.3)	110 (71.4)	0.023
Antenatal steroids	199 (57.5)	106 (55.2)	93 (60.4)	0.390
Pregnancy hypertension	61 (17.6)	30 (15.6)	31 (20.1)	0.342
Gestational diabetes	32 (9.2)	18 (9.4)	14 (9.1)	1.000
Chorioamnionitis	26 (7.5)	13 (6.8)	13 (8.4)	0.703
Resuscitation at birth	110 (31.8)	52 (27.1)	58 (37.7)	0.047
Endotracheal intubation^#^	58 (16.8)	28 (14.6)	30 (19.5)	0.286
Surfactant therapy^#^	104 (30.1)	47 (24.5)	57 (37.0)	0.016
Plastic wrap	148 (42.8)	79 (41.1)	69 (44.8)	0.566
Cap used	249 (72.0)	135 (70.3)	114 (74.0)	0.520
Preheated incubator^#^	230 (66.5)	133 (69.3)	97 (63.0)	0.264
Warm oxygen	165 (47.7)	97 (50.5)	68 (44.2)	0.285
Warmed fluids	123 (35.5)	67 (34.9)	56 (36.4)	0.865
Small for gestational age	45 (13.0)	21 (10.9)	24 (15.6)	0.264
Season				0.001
Autumn	75 (21.7)	44 (22.9)	31 (20.1)	
Spring	99 (28.6)	57 (29.7)	42 (27.3)	
Summer	79 (22.8)	55 (28.6)	24 (15.6)	
Winter	93 (26.9)	36 (18.8)	57 (37.0)	

Data are presented as mean ± standard deviation (SD), median (interquartile range, IQR), or number (percentage), as appropriate. Admission hypothermia was defined as an axillary temperature <36.5 °C at hospital admission. Continuous variables were compared using the Student’s *t*-test or the Mann–Whitney *U* test, as appropriate. Categorical variables were compared using the chi-square test or Fisher’s exact test. The *p*-value for season represents the overall comparison of seasonal distribution between groups. Ambient temperature refers to the outdoor ambient temperature at the receiving hospital at the time of admission (°C), time-matched to the admission timestamp, and does not represent delivery-room or transport-incubator internal temperature. Transport time was defined as minutes from departure from the birth/referring location to NICU admission. ^#^Variables retained in the final model following LASSO feature selection.

### Comparison of baseline characteristics between the training and validation cohorts

Following random allocation, 242 preterm infants were assigned to the training cohort and 104 to the validation cohort. As shown in Table 2, no statistically significant differences were observed between the two cohorts across most baseline demographic, perinatal, and clinical characteristics, indicating good baseline comparability. Gestational age, birth weight, maternal age, Apgar scores at 1 and 5 min, ambient temperature at admission, time to first temperature measurement, and the distribution of major perinatal interventions were similar between cohorts (all *p* > 0.05). A modest difference was observed in transport time, with the training cohort having a slightly longer median transport time than the validation cohort (25.0 vs. 21.0 min, *p* = 0.037). However, no significant between-cohort differences were noted for sex, multiple birth, mode of delivery, inborn status, antenatal steroid exposure, maternal comorbidities, resuscitation measures, or thermal care practices. In addition, the seasonal distribution of admissions did not differ significantly between the training and validation cohorts (*p* = 0.515). Overall, these findings support the adequacy of the random split and the representativeness of the validation cohort for subsequent model evaluation.

**Table 2:** Comparison of baseline characteristics between the training and validation cohorts.

Item	Overall (*n* = 346)	Training (*n* = 242)	Validation (*n* = 104)	*p*-value
Gestational age, weeks^#^	31.92 ± 2.51	31.91 ± 2.56	31.94 ± 2.38	0.916
Birth weight, g^#^	1863.50 (1488.25–2209.00)	1855.50 (1482.50–2192.75)	1891.50 (1536.50–2275.00)	0.500
Maternal age, years^#^	29.66 ± 4.72	29.87 ± 4.68	29.18 ± 4.81	0.215
Apgar score at 1 min	6.73 ± 1.67	6.78 ± 1.61	6.61 ± 1.81	0.371
Apgar score at 5 min	8.38 ± 1.61	8.42 ± 1.54	8.30 ± 1.77	0.515
Transport time, min^#^	24.00 (15.00–35.00)	25.00 (16.00–37.00)	21.00 (14.00–32.00)	0.037
Ambient temperature, °C^#^	17.99 ± 6.41	17.63 ± 6.48	18.84 ± 6.21	0.105
Time to first temperature measurement, min^#^	15.00 (11.00–19.75)	15.00 (11.00–20.00)	15.50 (10.00–19.00)	0.713
Male sex	179 (51.7)	124 (51.2)	55 (52.9)	0.870
Multiple birth^#^	82 (23.7)	54 (22.3)	28 (26.9)	0.432
Cesarean delivery	167 (48.3)	116 (47.9)	51 (49.0)	0.943
Inborn^#^	268 (77.5)	184 (76.0)	84 (80.8)	0.409
Antenatal steroids	199 (57.5)	145 (59.9)	54 (51.9)	0.207
Pregnancy hypertension	61 (17.6)	40 (16.5)	21 (20.2)	0.505
Gestational diabetes	32 (9.2)	20 (8.3)	12 (11.5)	0.446
Chorioamnionitis	26 (7.5)	19 (7.9)	7 (6.7)	0.889
Resuscitation at birth	110 (31.8)	79 (32.6)	31 (29.8)	0.694
Endotracheal intubation^#^	58 (16.8)	41 (16.9)	17 (16.3)	1.000
Surfactant therapy^#^	104 (30.1)	73 (30.2)	31 (29.8)	1.000
Plastic wrap	148 (42.8)	100 (41.3)	48 (46.2)	0.475
Cap used	249 (72.0)	177 (73.1)	72 (69.2)	0.541
Preheated incubator^#^	230 (66.5)	164 (67.8)	66 (63.5)	0.513
Warm oxygen	165 (47.7)	116 (47.9)	49 (47.1)	0.982
Warmed fluids	123 (35.5)	84 (34.7)	39 (37.5)	0.708
Small for gestational age	45 (13.0)	36 (14.9)	9 (8.7)	0.161
Season				0.515
Autumn	75 (21.7)	54 (22.3)	21 (20.2)	
Spring	99 (28.6)	70 (28.9)	29 (27.9)	
Summer	79 (22.8)	50 (20.7)	29 (27.9)	
Winter	93 (26.9)	68 (28.1)	25 (24.0)	

Data are presented as mean ± standard deviation (SD), median (interquartile range, IQR), or number (percentage), as appropriate. Continuous variables were compared between the training and validation cohorts using the Student’s t test or the Mann–Whitney *U* test, as appropriate. Categorical variables were compared using the chi-square test or Fisher’s exact test. This table was constructed to assess baseline comparability between the training and validation cohorts following a random split of the study population. ^#^ Variables retained in the final model following LASSO feature selection.

### Feature selection using LASSO regression

**Figure 1:**
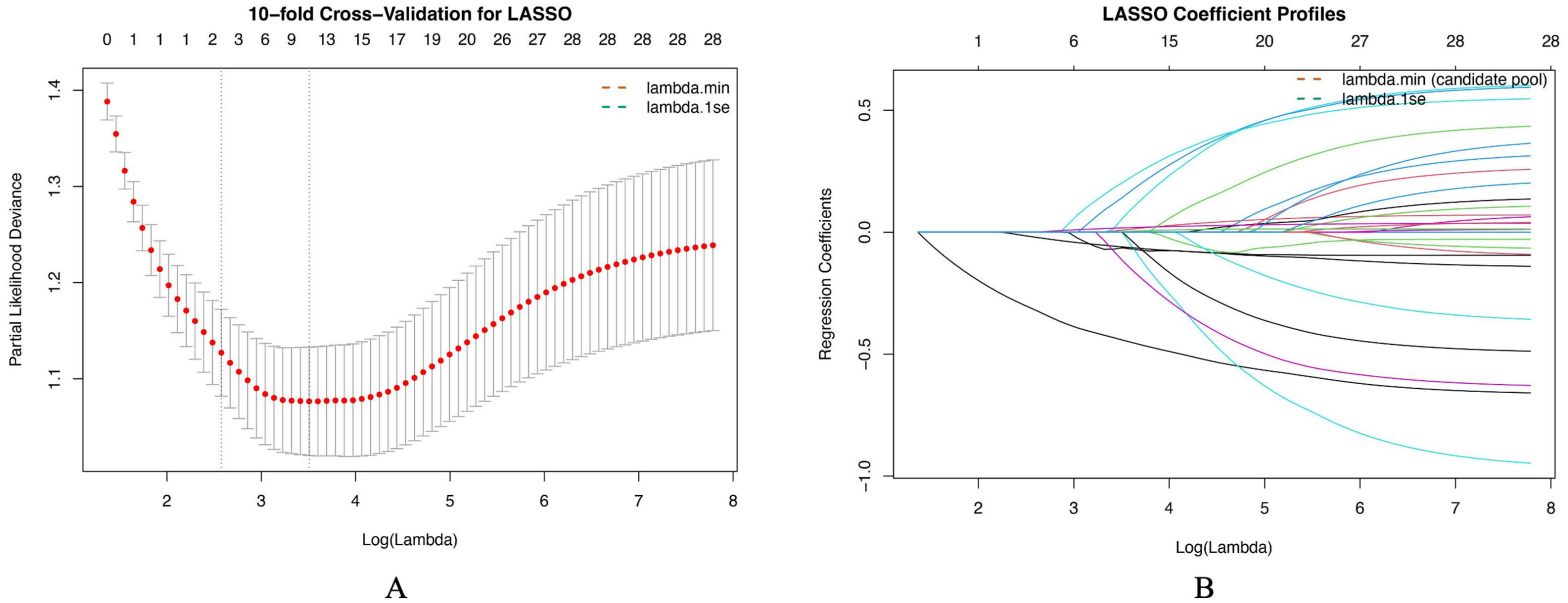
Feature selection using least absolute shrinkage and selection operator (LASSO) regression. **(A)** Cross-validated partial likelihood deviance plotted against log(*λ*). The dashed vertical lines indicate the optimal penalty parameter corresponding to the minimum mean cross-validated deviance (λmin) and the more parsimonious solution within one standard error of the minimum (λ1se). Numbers along the top axis represent the number of predictors with non-zero coefficients at each value of λ. **(B)** LASSO coefficient profiles for all candidate variables as a function of log(λ). Each curve represents the trajectory of an individual predictor’s regression coefficient. As the penalty parameter increases, coefficients are progressively shrunk toward zero. Variables with non-zero coefficients at λmin were retained as candidate predictors for subsequent model development.

Feature selection was performed using least absolute shrinkage and selection operator (LASSO) regression with 10-fold cross-validation to identify candidate predictors associated with admission hypothermia (Figure 1). Importantly, LASSO feature selection was performed only in the training cohort, and the validation cohort was not used for variable selection or model tuning. As shown in Figure 1A, the cross-validated partial likelihood deviance reached its minimum at the optimal penalty parameter *λ*min, while a more parsimonious solution was obtained at λ1se. To retain potentially informative variables for subsequent model development, predictors with non-zero coefficients at λmin were selected. The coefficient trajectories of individual variables across the range of log(λ) values are illustrated in Figure 1B, demonstrating progressive shrinkage of regression coefficients toward zero as the penalty increased. At λmin, a total of 11 variables were retained as candidate predictors, including gestational age, birth weight, maternal age, transport time, ambient temperature, time to first temperature measurement, multiple birth, inborn status, preheated incubator use, endotracheal intubation, and surfactant therapy (Supplementary Table S1). Among these variables, lower gestational age, lower birth weight, lower ambient temperature, non-inborn status, and absence of preheated incubator use were associated with an increased risk of admission hypothermia, as indicated by negative standardized coefficients for protective factors. In contrast, surfactant therapy, endotracheal intubation, multiple birth, and longer transport time showed positive coefficients, suggesting a higher risk of hypothermia. Collectively, these variables constituted the candidate feature set for subsequent machine learning–based risk prediction model construction.

### Model development and performance evaluation of machine learning–based prediction models

**Figure 2:**
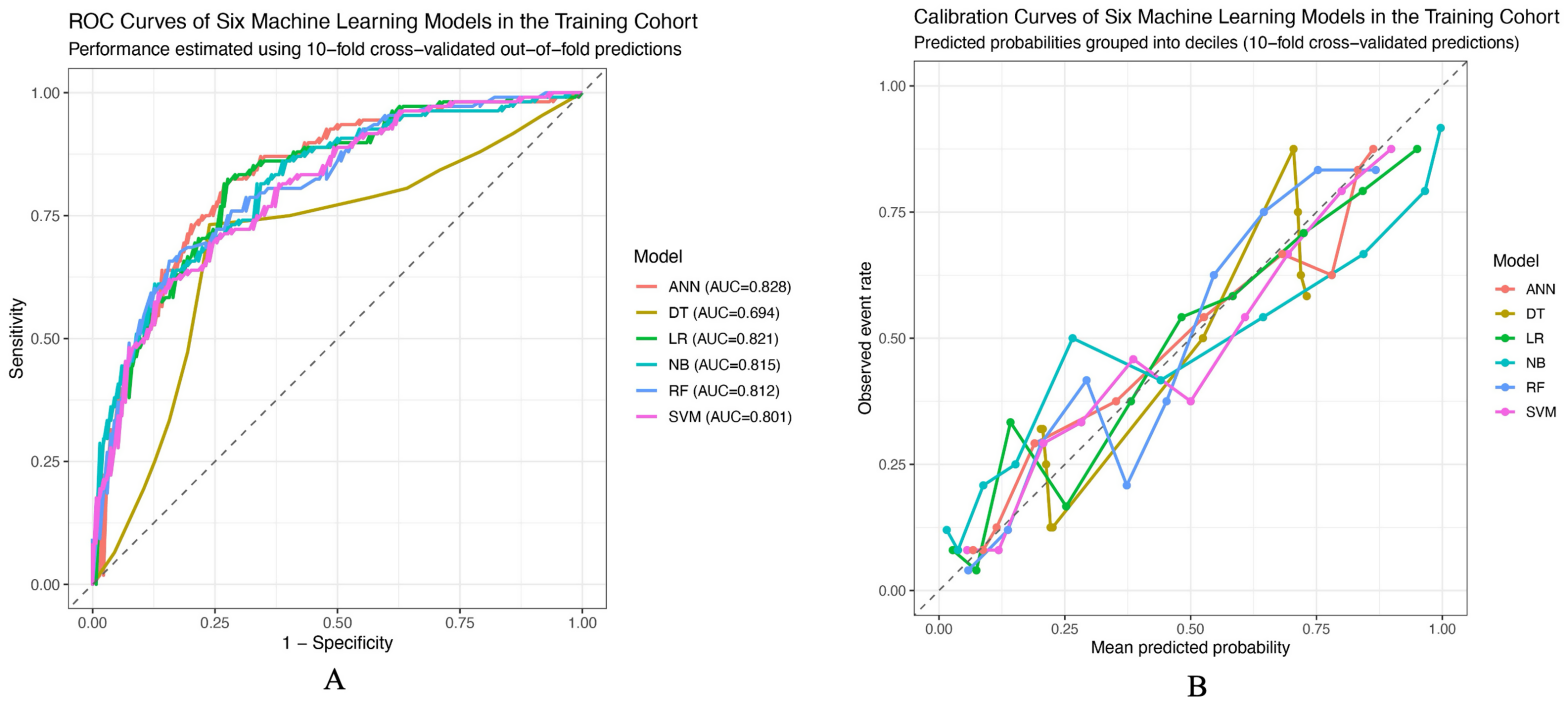
Discrimination and calibration of six machine learning models in the training cohort. **(A)** Receiver operating characteristic (ROC) curves comparing the discriminative ability of the six models. The diagonal dashed line represents random classification. The area under the ROC curve (AUC) for each model is shown in the legend. **(B)** Calibration curves depicting the agreement between predicted probabilities and observed event rates. Predicted risks were grouped into deciles based on out-of-fold predictions from 10-fold cross-validation. The dashed diagonal line indicates perfect calibration, where predicted probabilities equal observed outcomes. ANN, artificial neural network; DT, decision tree; LR, logistic regression; NB, naïve Bayes; RF, random forest; SVM, support vector machine.

**Figure 3:**
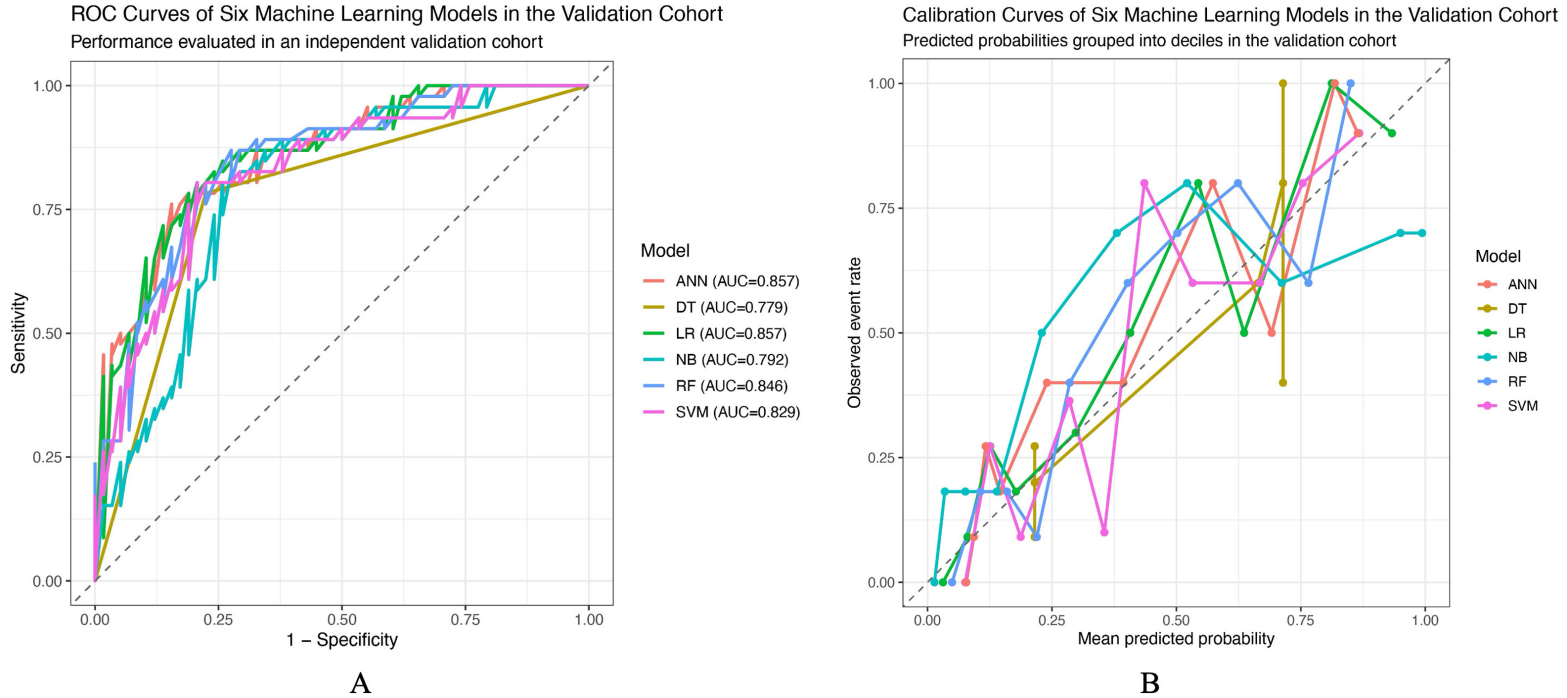
Discrimination and calibration of six machine learning models in the validation cohort. **(A)** Receiver operating characteristic (ROC) curves comparing the discriminative performance of the six models in the validation cohort. The diagonal dashed line represents random classification. The area under the ROC curve (AUC) for each model is displayed in the legend. **(B)** Calibration curves showing the agreement between predicted probabilities and observed event rates in the validation cohort. Predicted risks were grouped into deciles based on model-predicted probabilities. The dashed diagonal line indicates perfect calibration. ANN, artificial neural network; DT, decision tree; LR, logistic regression; NB, naïve Bayes; RF, random forest; SVM, support vector machine.

Based on the candidate predictors identified by LASSO regression at the optimal penalty parameter λmin (Supplementary Table S1), six machine learning models were developed and evaluated, including logistic regression (LR), decision tree (DT), random forest (RF), support vector machine (SVM), artificial neural network (ANN), and naïve Bayes (NB). Model performance in the training cohort was assessed using 10-fold cross-validated out-of-fold predictions, while an independent validation cohort was used to evaluate generalizability. In the training cohort, all six models demonstrated acceptable discriminative ability, with area under the receiver operating characteristic curve (AUC) values ranging from 0.69 to 0.83 (Table 3). Among them, the ANN model achieved the highest AUC (0.83), followed closely by LR (0.82), NB (0.82), and RF (0.81). The DT model showed comparatively lower discrimination (AUC = 0.69). Accuracy, sensitivity, specificity, and F1 scores were generally balanced across models, whereas RF and LR exhibited relatively favorable calibration performance, as reflected by lower Brier scores. The receiver operating characteristic curves for the training cohort are illustrated in Figure 2A, and calibration curves based on decile-grouped predicted probabilities are shown in Figure 2B, indicating reasonable agreement between predicted and observed risks across most probability ranges. In the validation cohort, overall model performance remained stable, with AUC values ranging from 0.78 to 0.86 (Table 3). Both LR and ANN achieved the highest discriminative performance (AUC = 0.86), followed by RF (AUC = 0.85) and SVM (AUC = 0.83). Although the DT model showed moderate discrimination (AUC = 0.78), its sensitivity was relatively high (0.783), whereas NB demonstrated comparatively lower discrimination and calibration performance, with the highest Brier score. The ROC curves in the validation cohort (Figure 3A) showed consistent separation from the reference line across models, while calibration curves (Figure 3B) suggested acceptable calibration for LR and ANN, with modest deviations observed for tree-based and probabilistic classifiers at higher predicted risk levels. Overall, models developed using the LASSO-selected feature set demonstrated consistent discrimination and calibration across training and validation cohorts. Considering the balance between discriminative ability, calibration performance, and clinical interpretability, logistic regression was selected as the final clinical model, whereas the other algorithms were evaluated primarily for comparative benchmarking rather than for direct bedside implementation. This benchmarking strategy also allowed us to assess whether more flexible models exhibited signs of optimism relative to LR under the same internal validation framework.

**Table 3:** Comparison of evaluation metrics for six machine learning models.

Cohort	Model	AUC	Accuracy	Sensitivity	Specificity	F1	Brier
Training cohort	LR	0.82	0.744	0.694	0.784	0.71	0.17
	DT	0.69	0.748	0.731	0.761	0.72	0.19
	RF	0.81	0.756	0.667	0.828	0.71	0.18
	SVM	0.8	0.727	0.694	0.754	0.69	0.18
	ANN	0.83	0.764	0.75	0.776	0.74	0.17
	NB	0.82	0.731	0.657	0.791	0.69	0.19
Validation cohort	LR	0.86	0.788	0.696	0.862	0.74	0.15
	DT	0.78	0.779	0.783	0.776	0.76	0.17
	RF	0.85	0.74	0.587	0.862	0.67	0.16
	SVM	0.83	0.731	0.609	0.828	0.67	0.17
	ANN	0.86	0.779	0.674	0.862	0.73	0.16
	NB	0.79	0.702	0.565	0.81	0.63	0.22

LR, logistic regression; DT, decision tree; RF, random forest; SVM, support vector machine; ANN, artificial neural network; NB, naïve Bayes. Model performance in the training cohort was evaluated using 10-fold cross-validation, with reported metrics calculated from out-of-fold predictions. Performance in the validation cohort was assessed using an independent validation dataset. The area under the receiver operating characteristic curve (AUC) was used to assess discriminative ability. Accuracy, sensitivity, specificity, and F1 score were calculated using the optimal cutoff determined in the training cohort. The Brier score reflects overall calibration performance, with lower values indicating better agreement between predicted probabilities and observed outcomes.

### SHAP-based interpretation of model predictions

**Figure 4:**
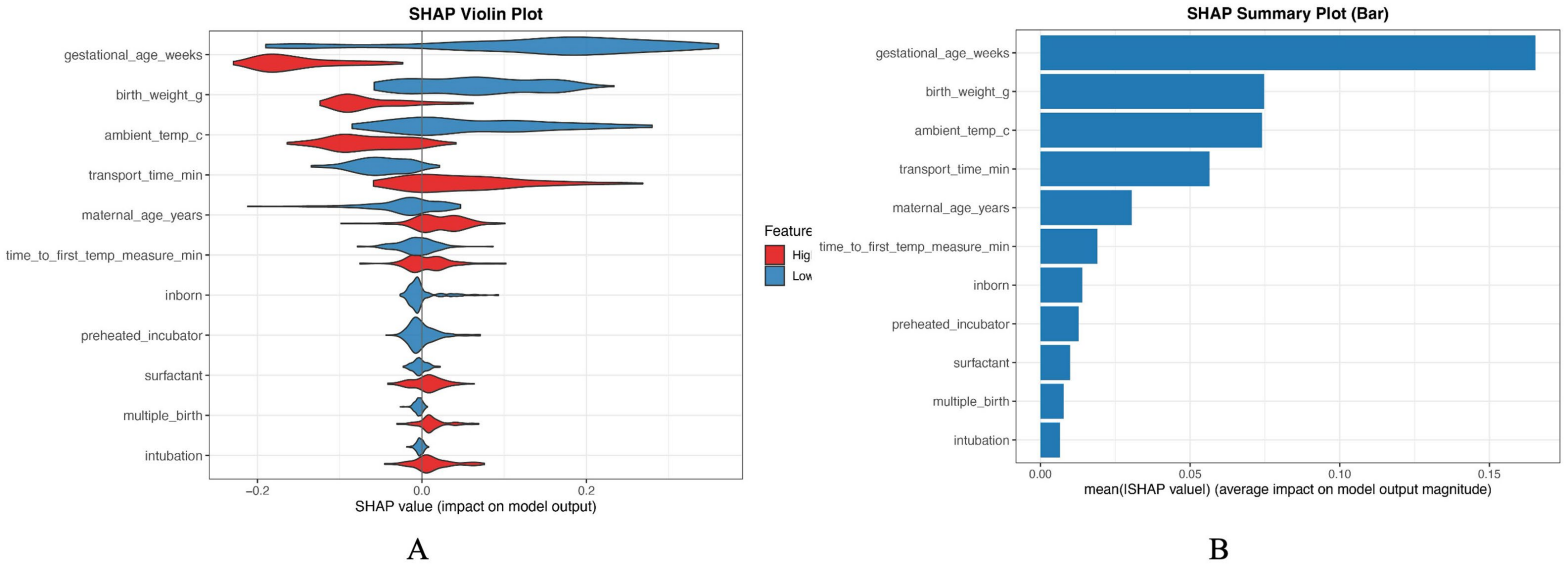
SHAP-based interpretation of the prediction model using a random forest surrogate. **(A)** SHAP violin plot showing the distribution of SHAP values for each predictor across all infants. Each point represents an individual observation, with color indicating feature value (red = high, blue = low). Positive SHAP values indicate an increased predicted risk of admission hypothermia, whereas negative values indicate a protective effect. **(B)** SHAP summary bar plot ranking predictors according to their mean absolute SHAP values, reflecting the overall importance of each variable in the model output. Larger values indicate greater influence on the prediction. Logistic regression was selected as the final clinical model; RF was used only as a surrogate to derive SHAP explanations.

To enhance interpretability and to explore potential non-linear effects and feature interactions, we performed SHAP (Shapley Additive Explanations) analysis using a random forest (RF) surrogate model trained on the same LASSO-selected predictors (Figure 4). Although logistic regression (LR) was selected as the final model for clinical application due to its favorable balance between discrimination, calibration, and transparency, RF was used only for SHAP-based explanation because tree-based models provide an efficient and stable SHAP implementation and are well-suited to capturing non-linear relationships and interactions. The SHAP summary bar plot (Figure 4B) ranks predictors according to their mean absolute SHAP values, reflecting their overall contribution to the model output. Gestational age was identified as the most influential predictor, followed by birth weight, ambient temperature at admission, and transport time, underscoring the predominant role of neonatal maturity and environmental exposure in determining admission hypothermia risk. Maternal age and time to first temperature measurement showed moderate contributions, whereas inborn status, preheated incubator use, surfactant therapy, multiple birth, and endotracheal intubation contributed relatively less to the overall prediction. The SHAP violin plot (Figure 4A) further illustrates the direction and distribution of each predictor’s effect. Lower gestational age and lower birth weight were associated with positive SHAP values, indicating an increased predicted risk of admission hypothermia, whereas higher values exerted protective effects. Similarly, lower ambient temperature and longer transport time were associated with higher predicted risk. In contrast, inborn status and the use of a preheated incubator tended to shift SHAP values toward the negative range, suggesting protective associations. For binary clinical variables such as surfactant therapy, endotracheal intubation, and multiple birth, positive SHAP values were mainly observed when these conditions were present, likely reflecting underlying illness severity rather than direct causal effects. Overall, the SHAP analysis revealed coherent and clinically plausible relationships between predictors and admission hypothermia risk, providing complementary interpretative insight that supports the face validity and robustness of the final logistic regression–based prediction model. Importantly, RF–SHAP was used solely for interpretability and did not affect model selection; the key predictors identified by SHAP were consistent with the LR-based findings.

## Discussion

In this retrospective cohort study, we developed and internally validated multiple machine learning–based models to predict admission hypothermia in preterm infants using routinely available perinatal, environmental, and transport-related variables. Admission hypothermia was observed in 44.5% of the study population, highlighting that hypothermia remains highly prevalent despite standardized neonatal resuscitation and thermal care protocols. Among the evaluated models, logistic regression and artificial neural network demonstrated the highest discriminative performance in the validation cohort, while logistic regression showed the most favorable balance between discrimination, calibration, and clinical interpretability.

The observed incidence of admission hypothermia in our cohort is consistent with previous reports from both high-income and middle-income settings, where prevalence rates ranging from 30% to over 50% have been described, particularly among very preterm and very low birth weight infants (18–20). This persistent burden underscores the clinical relevance of early risk stratification and supports the need for prediction tools that extend beyond guideline-based universal interventions. Our findings further reinforce existing evidence that admission hypothermia is a multifactorial condition influenced by neonatal immaturity, environmental exposure, and perinatal care processes rather than a single modifiable factor (21).

Gestational age and birth weight emerged as the most influential predictors in both traditional modeling and SHAP-based interpretation, aligning with well-established physiological mechanisms. Preterm infants with lower gestational age and birth weight have immature skin barriers, reduced brown adipose tissue, and limited metabolic capacity for heat production, rendering them particularly susceptible to heat loss immediately after birth (22, 23). Environmental factors, especially lower ambient temperature at admission and prolonged transport time, were also strongly associated with increased hypothermia risk. These findings are consistent with prior observational studies demonstrating that thermal instability during transport and suboptimal environmental control are key contributors to admission hypothermia, particularly for outborn infants (24).

Notably, inborn status and the use of a preheated incubator were identified as protective factors, as reflected by negative SHAP values. This observation is in line with previous reports suggesting that delivery within tertiary centers and timely application of active thermal care measures can reduce the risk of hypothermia (25). Conversely, clinical interventions such as surfactant therapy and endotracheal intubation were associated with higher predicted risk. These variables likely serve as proxies for underlying illness severity and physiological instability rather than direct causal determinants of hypothermia, a distinction that is important for appropriate clinical interpretation (26).

Compared with prior studies that primarily relied on univariable or multivariable regression to identify risk factors, the present study extends the literature by systematically comparing multiple machine learning approaches and integrating feature selection, internal validation, and model interpretation within a unified framework. The rationale for evaluating multiple machine learning algorithms was to provide a transparent benchmark comparison and to test whether non-linear methods offered clinically meaningful incremental predictive value beyond LR in this structured dataset. Although logistic regression was selected as the final clinical model, SHAP analysis was performed using a random forest surrogate to explore potential non-linear relationships and interactions; importantly, the key predictors identified through SHAP were consistent with those derived from the logistic regression model. While more complex models such as random forest and artificial neural networks demonstrated competitive discriminative performance, logistic regression achieved comparable accuracy with superior interpretability and stable calibration. This finding supports previous methodological research suggesting that, in structured clinical datasets, simpler models may perform similarly to complex algorithms while offering greater transparency and ease of implementation (27).

The clinical applicability of this work lies in providing a simple, bedside-ready risk stratification tool that uses routinely available variables (e.g., gestational age, birth weight, ambient temperature, inborn status, and expected transport duration). In routine practice, the transfer team can estimate transport duration at the start of transfer using the referring site and historical transfer times, enabling risk stratification before transport-related thermal exposure occurs. In practice, the model is intended for use at two actionable timepoints: (i) before departure/early during transfer using routinely known perinatal variables plus an estimated transport duration, and (ii) at NICU reception using the observed admission workflow variables, to support risk-triggered escalation of thermoprotection. Infants classified as high risk could trigger protocolized escalation of thermal protection, such as prioritizing a preheated incubator for transport, minimizing exposure time during handover, ensuring plastic wrap and cap use when appropriate, using warmed and humidified respiratory support, warming fluids/oxygen when indicated, and implementing earlier and more frequent temperature checks during transport and the first minutes after admission. By flagging high-risk infants in advance, the model may help teams standardize vigilance and prioritize limited thermal care resources in busy units. Importantly, this model is intended to complement—not replace—guideline-based thermoregulation bundles, serving as a decision-support layer to target intensified measures to infants most likely to benefit. Given its transparency and ease of implementation, LR is more likely to be accepted and audited in routine NICU workflows than black-box models when predictive performance is similar.

To support realistic deployment in a busy NICU, we envision two pragmatic pathways. First, a bedside ‘quick-entry’ approach can be used at the point of admission or before transfer: gestational age and birth weight are routinely available immediately after birth, inborn status is known *a priori*, and ambient temperature can be entered from the receiving-site weather monitoring display; transport duration can be entered as an estimated value based on the referring location and standard transfer workflow. This enables rapid risk stratification (typically within minutes) and can trigger pre-specified escalation steps such as prioritizing a preheated transport incubator, minimizing exposure during handover, ensuring plastic wrap and cap use when appropriate, using warmed/humidified respiratory support if indicated, and increasing the frequency of temperature checks during transfer and immediately after NICU arrival. Second, an automated EHR-embedded option could compute risk in the background once the same variables are entered routinely, generating a non-intrusive flag within existing admission/transport documentation rather than interruptive pop-up alerts. Evidence syntheses on deployed prediction tools emphasize that clinical value depends less on discrimination alone and more on how the model is embedded (e.g., interruptive vs. non-interruptive displays), how thresholds are selected, and whether teams invest in training, governance, and ongoing performance monitoring—without which implementation may fail or be abandoned in routine care (28). Potential barriers include incomplete or delayed documentation (particularly for transport variables), heterogeneity in local transport protocols and thermoregulation bundles, staff workload and training needs, and alert fatigue if thresholds are not carefully tuned. Importantly, real-world implementation studies of EHR-embedded predictive models consistently highlight sociotechnical barriers—particularly alert fatigue, insufficient user training, and increased workload—that can attenuate the clinical impact of otherwise well-performing models (29). In the NICU, the high density of monitoring devices and frequent nonactionable alarms can desensitize staff and prolong response times, underscoring that any risk-flagging tool must be designed to minimize unnecessary signaling to avoid compounding alarm fatigue (30). Finally, because calibration may drift across climates and workflows, local validation and recalibration (intercept/slope adjustment) should be performed prior to routine clinical use. Accordingly, a sociotechnical systems perspective suggests that successful adoption requires alignment with the broader work system (including roles, handoffs, documentation processes, and workflow timing), because a predictive model is only one component of a complex clinical intervention that must fit the surrounding environment to achieve reliable benefit (31).

Several limitations merit consideration. First, this was a single-center retrospective study, which may limit generalizability to other neonatal units with different patient populations, transport systems, or thermal care practices. Second, although we performed internal validation using both cross-validation and an independent split-sample validation cohort, external validation in geographically and operationally distinct neonatal units is required to confirm transportability. This is particularly important for more flexible models (e.g., ANN/RF/SVM), which can overfit idiosyncratic single-center patterns and yield optimistic discrimination when evaluated in a split from the same dataset. External validation is essential because both discrimination and calibration may shift across settings due to differences in case-mix (e.g., gestational age distribution and illness severity), thermal care and transport workflows, temperature measurement timing/devices, and local climate/seasonality. Multicenter validation therefore evaluates transportability and informs whether recalibration or model updating is required prior to implementation. Accordingly, our findings should be interpreted as internal validation evidence supporting feasibility within the study setting, rather than definitive proof of cross-center generalizability. Because the model was developed using recorded (observed) transport time, future prospective studies should validate performance when substituting pre-transport estimated duration to reflect the earliest actionable decision point. Third, we did not have granular measurements of delivery-room temperature or transport-incubator internal temperature (or temperature trajectories during transport); therefore, we used outdoor ambient temperature at admission as a pragmatic environmental proxy. Future prospective studies should capture these more proximal thermal exposure measures to further refine model transportability. Additionally, although admission temperature is routinely documented as part of standardized NICU admission procedures, a small proportion of infants were excluded due to missing outcome data. While chart review did not suggest systematic enrichment of extreme illness severity among these cases, residual selection bias related to missing documentation cannot be entirely excluded. Finally, as with all observational studies, the identified associations should not be interpreted as causal relationships.

In conclusion, this study demonstrates that machine learning–based models using routinely available clinical and environmental data can effectively predict admission hypothermia in preterm infants. Logistic regression provided robust performance with favorable interpretability, supporting its potential utility for early risk stratification in neonatal clinical practice. Future multicenter studies with external validation are warranted before routine clinical use to confirm transportability and to explore integration of the model into real-world neonatal care workflows. From an implementation perspective, the LR model could be translated into a simple bedside score or embedded into the electronic medical record as an automated risk alert using routinely entered admission variables.

## Data Availability

The original contributions presented in the study are included in the article/Supplementary material, further inquiries can be directed to the corresponding author.
