# Supplementary material for: Development and internal validation of machine learning–based models for predicting admission hypothermia in preterm infants: a retrospective cohort study
Source: Front Med (Lausanne). 2026 Mar 25;13:1811064. doi: 10.3389/fmed.2026.1811064 (PMC13057518; doi:10.3389/fmed.2026.1811064)
Supplement: Supplementary file 1 [file Table_1.docx]

**Supplementary Table S1.** Candidate Predictors Selected by LASSO Regression at λmin

| **Variable** | **Coefficient** |
| --- | --- |
| Gestational age (weeks) | −0.4419 |
| Surfactant therapy | 0.2022 |
| Endotracheal intubation | 0.1414 |
| Preheated incubator | −0.1152 |
| Inborn | −0.0642 |
| Ambient temperature (°C) | −0.0603 |
| Multiple birth | 0.0557 |
| Transport time (min) | 0.0177 |
| Maternal age (years) | 0.0086 |
| Time to first temperature measurement (min) | 0.0027 |
| Birth weight (g) | −0.0001 |

**Note:** Candidate predictors were selected using the least absolute shrinkage and selection operator (LASSO) regression with 10-fold cross-validation. The penalty parameter was chosen based on the minimum cross-validated deviance (λmin). Variables with non-zero coefficients at λmin were retained as candidate predictors for subsequent model development. Coefficients represent standardized regression coefficients from the LASSO model; a positive coefficient indicates an increased risk of admission hypothermia, whereas a negative coefficient indicates a protective association.
